# Studies of the Association of Arg72Pro of Tumor Suppressor Protein p53 with Type 2 Diabetes in a Combined Analysis of 55,521 Europeans

**DOI:** 10.1371/journal.pone.0015813

**Published:** 2011-01-20

**Authors:** Kristoffer Sølvsten Burgdorf, Niels Grarup, Johanne Marie Justesen, Marie Neergaard Harder, Daniel Rinse Witte, Torben Jørgensen, Annelli Sandbæk, Torsten Lauritzen, Sten Madsbad, Torben Hansen, DIAGRAM Consortium, Oluf Pedersen

**Affiliations:** 1 Hagedorn Research Institute, Copenhagen, Denmark; 2 Hvidovre Hospital, Copenhagen, Denmark; 3 Steno Diabetes Center, Copenhagen, Denmark; 4 Research Centre for Prevention and Health, Glostrup University Hospital, Glostrup, Denmark; 5 Faculty of Health Sciences, Institute of Biomedical Science, University of Copenhagen, Copenhagen, Denmark; 6 Department of General Practice, Institute of Public Health, Aarhus University, Aarhus, Denmark; 7 Faculty of Health Sciences, University of Southern Denmark, Odense, Denmark; 8 Faculty of Health Science, University of Aarhus, Aarhus, Denmark; Peninsula College of Medicine and Dentistry, University of Exeter, United Kingdom

## Abstract

**Aims:**

A study of 222 candidate genes in type 2 diabetes reported association of variants in *RAPGEF1*, *ENPP1*, *TP53*, *NRF1*, *SLC2A2*, *SLC2A4* and *FOXC2* with type 2 diabetes in 4,805 Finnish individuals. We aimed to replicate these associations in a Danish case-control study and to substantiate any replicated associations in meta-analyses. Furthermore, we evaluated the impact on diabetes-related intermediate traits in a population-based sample of middle-aged Danes.

**Methods:**

We genotyped nine lead variants in the seven genes in 4,973 glucose-tolerant and 3,612 type 2 diabetes Danish individuals. In meta-analyses we combined case-control data from the DIAGRAM+ Consortium (n = 47,117) and the present genotyping results. The quantitative trait studies involved 5,882 treatment-naive individuals from the Danish Inter99 study.

**Results:**

None of the nine investigated variants were significantly associated with type 2 diabetes in the Danish samples. However, for all nine variants the estimate of increase in type 2 diabetes risk was observed for the same allele as previously reported. In a meta-analysis of published and online data including 55,521 Europeans the G-allele of rs1042522 in *TP53* showed significant association with type 2 diabetes (OR = 1.06 95% CI 1.02–1.11, *p* = 0.0032). No substantial associations with diabetes-related intermediary phenotypes were found.

**Conclusion:**

The G-allele of *TP53* rs1042522 is associated with an increased prevalence of type 2 diabetes in a combined analysis of 55,521 Europeans.

## Introduction

Type 2 diabetes is a rapidly growing health problem worldwide. Although the epidemic nature of the disease may be attributable mainly to environmental factors leading to obesity, also genetic factors predispose to the disease. Type 2 diabetes is characterized by insulin resistance in skeletal muscle, liver and adipose tissue, in combination with insufficient pancreatic beta-cell function, leading to elevated levels of blood glucose.

In the past three years numerous genome-wide association studies (GWAS) have identified common type 2 diabetes susceptibility variants [1]. Although GWAS have proved superior to candidate gene based approaches in the discovery of susceptibility genes for complex diseases there may still be a need for well-designed targeted evaluation of disease candidate genes. A publication in 2008 used a complementary approach by performing an in-depth, step-wise analysis of 222 candidate genes likely to influence susceptibility to type 2 diabetes [2], selected by applying CandidAtE Search And Rank (CAESAR), a text- and data mining algorithm [3]. In a case-control study of 1,161 type 2 diabetic patients and 1,174 control subjects, 3,531 variants in these 222 genes were genotyped and after imputation of additional variants, coverage of 99.9% of common HapMap variants was achieved. Selected single-nucleotide polymorphisms (SNPs) were genotyped in additional 1,211 type 2 diabetic cases and 1,259 control individuals. Of the investigated variants, 16 were associated with type 2 diabetes at a 5% significance level in the combined study sample [2]. The top ten type 2 diabetes variants included variants in eight genes: RAPGEF1 rs4740283, ENPP1 rs2021966 and rs858341, NRF1 rs1882095, TP53 rs1042522, SLC2A2 rs10513684 and rs5400, SLC2A4 rs222852, FOXC2 rs4843165, and PPARG.

The variant in PPARG is a confirmed type 2 diabetes polymorphism at genome-wide significance level [4], while the rest are either novel putative type 2 diabetes susceptibility loci or variants in genes previously associated with diabetes risk at a conventional significance level. In the present report, we aimed at replicating the top nine novel associations with type 2 diabetes in seven loci in a Danish case-control study (excluding the previously investigated *PPARG* variant) followed by a meta-analysis of our data and reported association studies. Furthermore, we evaluated the impact of these variants on diabetes-related intermediate traits in the Inter99 cohort where study participants have been characterized by an oral glucose tolerance test (OGTT) and derived estimates of insulin release and insulin sensitivity.

## Materials and Methods

### Participants

The studies were approved by the regional Ethical Committees and were in accordance with the principles of the Helsinki Declaration. The nine variants were genotyped in 5,882 treatment-naive middle-aged individuals from the Inter99 cohort and in additional Danish individuals totalling a case-control study of 4,973 glucose-tolerant participants and 3,612 type 2 diabetic cases. The total of 10,157 Danish individuals were ascertained from four different study groups; 1) the Inter99 cohort which is a randomized, non-pharmacological intervention study of randomly ascertained middle-aged individuals for the prevention of ischemic heart disease, conducted at the Research Centre for Prevention and Health in Glostrup, Copenhagen (ClinicalTrials.gov ID-no: NCT00289237) [5], [6] (*n* = 5,999); 2) type 2 diabetic patients from the Danish Anglo-Danish-Dutch Study of Intensive Treatment in People with Screen-Detected Diabetes in Primary Care (ADDITION) study (ClinicalTrials.gov ID-no: NCT00237548) [7], which is a population-based, high-risk screening and intervention study for type 2 diabetes in general practice (*n* = 1,583); 3) Type 2 diabetes patients and normal glucose tolerant subjects from a randomly recruited group of unrelated middle-aged individuals (*n* = 562) examined at Steno Diabetes Center; and 4) unrelated type 2 diabetic patients (*n* = 2,013) sampled through the out-patient clinic at Steno Diabetes Center. All participants in study group 1 and 3 underwent a standard 75 g OGTT. Type 2 diabetes and glucose tolerance status were defined according to the World Health Organization 1999 criteria [8]. All study participants were Danes by self-report and informed written consent was obtained from all before participation. An overview of the clinical characteristics of the four study groups is given in Table S2. Description of the DIAGRAM+ study participants are described in the primary study by Voight et al [9].

### Biochemical and anthropometric measures

In all four study groups body weight and height were measured in light indoor clothes and without shoes. BMI was defined as weight in kilograms divided by height in meters squared (kg/m^2^). Waist circumference (cm) was measured with subjects in standing position midway between the iliac crest and the lower costal margin. Blood samples were drawn after a 12 h overnight fast. Plasma glucose was analysed by a glucose oxidase method (Granutest; Merck, Darmstadt, Germany). HbA_1c_ was measured by ion-exchange high-performance liquid chromatography (normal reference range: 4.1–6.4%) and serum insulin, excluding des(31, 32) and intact proinsulin, was measured using an insulin kit (AutoDELFIA, Perkin-Elmer/Wallac, Turku, Finland). Serum C-peptide concentrations were measured by a time-resolved fluoroimmunoassay (AutoDELFIA C-peptide kit; Perkin-Elmer/Wallac). Serum triglyceride, total cholesterol and HDL-cholesterol were analysed using enzymatic colorimetric methods (GPO-PAP and CHOD-PAP, Roche Molecular Biochemicals, Basel, Switzerland). Homeostasis model assessment of insulin resistance (HOMA-IR) was calculated as described [10]. The OGTT-derived indices of insulin sensitivity (BIGTT-S*_I_*) and beta-cell function (BIGTT-acute insulin response (AIR)) were calculated as reported [11].

### Genotyping

Genotyping of the nine variants in 10,157 Danish individuals was done using KASPar® SNP genotyping (KBioscience, Herts, UK). All genotyping success rates were >95% and error rates were <0.4% estimated from re-genotyping of 513 random duplicate samples. Genotype distributions for all variants obeyed Hardy-Weinberg equilibrium in the investigated study groups (*P*>0.05).

### Statistical analysis

The case-control study of type 2 diabetes was performed by logistic regression. A *P*-value and odds-ratio were calculated for all variants, applying an additive model. Furthermore, we analysed the variants using the same models as in the original work by Gaulton et al. [2]. All studies were performed with and without adjusting for sex, age and BMI. Combined analyses of the present and previous studies were performed by fixed-effect, additive-models inverse variance-weighted meta-analysis. To test quantitative traits for differences between genotype groups a general linear model was used, applying additive models for each variant and including adjustments for sex and age (for waist, waist-to-hip ratio and BMI) or age, sex and BMI (all other traits). All analyses were performed by RGui version 2.8.0 [12], and *P-*values below a Bonferroni corrected (9 SNPs) threshold of 0.05/9 = 0.0056 were considered significant. Therefore, given the multiple quantitative phenotypes tested the analyses of diabetes-related intermediary traits are merely explorative. Homogeneity between studies was tested using the Mantel–Haenszel method with a generalised linear model. All meta-analyses were performed using an additive model.

Statistical power in case-control settings and meta-analyses was determined using the CaTS power calculator version 0.0.2. The prevalence of type 2 diabetes was estimated as 6%, providing 94% statistical power to detect associations with type 2 diabetes in the Danish study population and more than 99% in the meta-analyses, for a variant with a minor allele frequency of 10% and with a relative risk of 1.15.

## Results

### Association with type 2 diabetes

Each of the nine variants was analysed in a case-control study of type 2 diabetes including 3,612 Danish cases and 4,973 control subjects (Table 1). In order to replicate the previous findings by Gaulton et al., we used the same model for each variant in the case-control studies. Results for the additive models are also reported for all variants (Table 1). None of the nine investigated variants were significantly associated with type 2 diabetes after correction for multiple testing (all *P*>0.0056). However, for all nine variants the type 2 diabetes risk was non-significantly increased for the same allele as previously reported (Table 1).

**Table 1:** Type 2 diabetes association for nine gene variants as listed below and which were genotyped in 3,612 type 2 diabetes cases and 4,973 control individuals with normal glucose tolerance.

SNP	Gene symbol	Risk/non risk allele	Risk allele frequency	Model*	*P* value	Odds ratio (95% CI)	*P* value Additive
**rs2021966**	*ENPP1*	A/G	0.53	REC	0.0097	1.15 (1.03–1.28)	0.015
**rs858341**	*ENPP1*	G/A	0.38	REC	0.24	1.05 (0.95–1.22)	0.15
**rs4843165**	*FOXC2*	C/T	0.73	ADD	0.76	1.01 (0.94–1.08)	0.76
**rs1882095**	*NRF1*	T/C	0.36	DOM	0.15	1.07 (0.98–1.17)	0.26
**rs4740283**	*RAPGEF1*	G/A	0.14	REC	0.69	1.08 (0.78–1.49)	0.41
**rs10513684**	*SLC2A2*	C/T	0.94	ADD	0.37	1.06 (0.93–1.23)	0.37
**rs5400**	*SLC2A2*	G/A	0.87	ADD	0.17	1.06 (0.97–1.16)	0.17
**rs222852**	*SLC2A4*	A/G	0.60	ADD	0.45	1.02 (0.96–1.09)	0.45
**rs1042522**	*TP53*	G/C	0.27	ADD	0.094	1.06 (0.99–1.14)	0.094

P-value and odds ratio were calculated assuming the same genetic model as used in the study by Gaulton et al. [Gaulton2008] (REC = recessive, ADD = additive and DOM = dominant) and assuming additive model for all studies. Data are adjustet for sex age and BMI.

**Figure 1:**
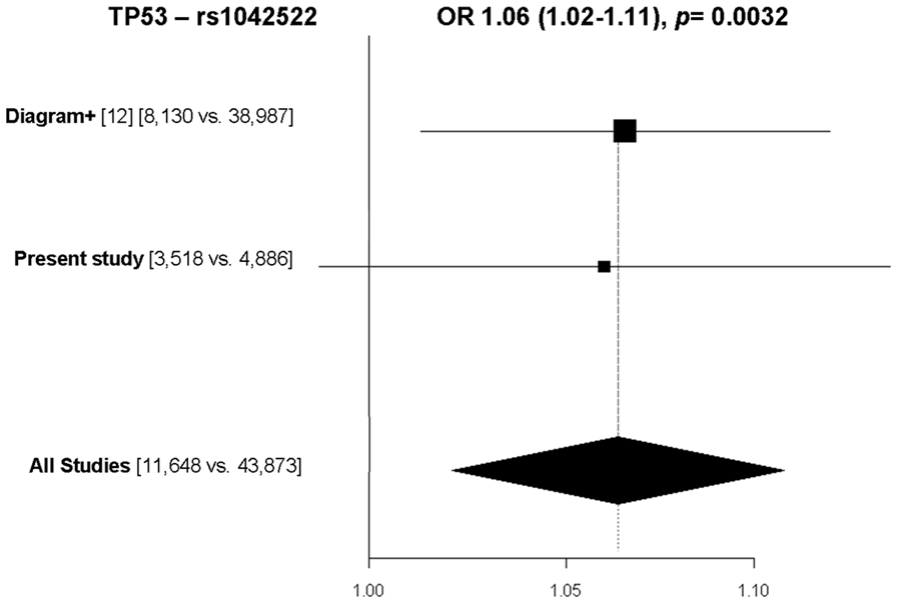
Estimated odd ratio (95% CIs) of present and previously reported associations of the G-allele of *TP53* rs1042522 with type 2 diabetes when analysed according to an additive genetic model (*n* = 55,521). No heterogeneity between studies was observed (*P* = 0.91). Numbers in square brackets designate numbers of type 2 diabetic patients and control subjects, respectively.

To further investigate the impact of the variants on type 2 diabetes risk, we performed combined analyses with data from DIAGRAM+ Consortium in relation to type 2 diabetes (8,130 cases and 38,987 control subjects) [9]. The meta-analysis included 55,521 individuals (11,648 cases and 43,873 control subjects). There were no heterogeneity between the study populations (*p* = 0.09–1.0) using the Mantel–Haenszel method with a generalised linear model. Applying an additive genetic model one of the nine variants, the minor G-allele of *TP53* rs1042522 was associated with type 2 diabetes in the combined analysis of all data: OR = 1.06 95% CI 1.02–1.11 *p* = 0.0032). (Figure 1).

Two of the remaining nine variants showed nominal association with type 2 diabetes in the meta-analyses: *ENPP1* rs2021966 (OR = 1.05, 95% CI 1.01–1.10, *P* = 0.012), and *ENPP1* rs5400 (OR 1.07 95% CI 1.01–1.12 *P* = 0.011).

### Analyses of quantitative metabolic traits

We investigated the relationship of the nine variants with quantitative diabetes-related metabolic traits in the Inter99 cohort of 5,772 treatment-naive individuals. Applying an additive genetic model the minor diabetes-associated G-allele of the *TP53* rs1042522 showed nominal association with higher levels of fasting plasma glucose (*P* = 0.03) (Table 2). In addition, the G-allele of *ENPP1* rs858341 was associated with higher plasma glucose at 30 min. post OGTT (*P* = 0.0022) and a larger area under the plasma glucose curve during the OGTT (*P* = 0.0022).

**Table 2:** Anthropometric and metabolic characteristics of 5,772 middle-aged treatment-naive Danish Inter99 participants stratified according to *TP53* rs1042522 genotype.

*TP53* rs1042522	CC	CG	GG	*P*
*n* (men/women)	3064 (1518/1546)	2283 (1137/1146)	425 (219/206)	
Age (years)	46±8	46±8	46±8	
BMI (kg/m^2^)	26.2±4.6	26.1±4.5	26.3±4.5	0.6
Waist-to-hip ratio	0.86±0.09	0.85±0.09	0.86±0.09	0.6
Waist (cm)	87±13	86±13	87±13	0.3
**Plasma glucose**				
Fasting (mmol/l)	5.5±0.8	5.5±0.8	5.6±1.2	0.03
30-min post-OGTT (mmol/l)	8.7±1.9	8.7±1.9	8.8±2	0.6
120-min post-OGTT (mmol/l)	6.2±2.1	6.2±2.1	6.5±2.4	0.09
Post-OGTT AUC (min⋅mmol/l)	220±134	220±137	229±139	0.5
**Serum insulin**				
Fasting (pmol/l)	43±28	41±27	44±31	0.7
30-min post-OGTT (pmol/l)	290±179	290±187	298±200	0.6
120-min post-OGTT (pmol/l)	217±216	213±202	237±238	0.06
Post-OGTT AUC (min⋅pmol/l)	22839±15921	22685±15451	24474±18572	0.1
HOMA-IR (mmol/l⋅pmol/l)	10.7±8.1	10.3±7.6	11.4±9.7	1
Insulinogenic index (pmol×pmol^−1^)	29±19	29±20	30±20	0.6
BIGTT-SI	9.2±4	9.3±4	8.9±4	0.8
BIGTT-AIR	1861±1099	1807±973	1919±1424	0.7
**Fasting serum lipids**				
Triglyceride (mmol/l)	1.3±1	1.4±1.7	1.4±1.2	0.1
Total cholesterol (mmol/l)	5.5±1.1	5.5±1.1	5.5±1.1	0.7
HDL-cholesterol (mmol/l)	1.4±0.4	1.4±0.4	1.4±0.4	0.4

Data are mean +/− standard deviation. Values of serum insulin, values derived from insulin variables, and values of serum triglyceride were logarithmically transformed before statistical analysis. Calculated *P* values were adjusted for age, sex, and for BMI (except BMI, waist-to-hip and waist), and were calculated assuming an additive model. HOMA-IR was calculated as fasting plasma glucose (mmol/l) multiplied by fasting serum insulin (pmol/l) and divided by 22.5. AUC, area under the curve.

Several other variants showed sporadic nominal associations with the investigated traits (Tables S3, S4, S5, S6, S7, S8, S9, S10). Furthermore, all variants were analysed using the same genetic model as in the primary study, but none of the variants gave stronger association with the quantitative traits.

## Discussion

In the present study we evaluated the evidence for association with type 2 diabetes for the nine most significantly associated novel candidate variants reported in a paper by Gaulton and colleges [2). None of them were associated with type diabetes in the Danish study samples. However, in the meta-analyses of 55,521 Europeans *TP53* rs1042522 remained significantly associated with type 2 diabetes after correction for multiple testing. Thus, *TP53* rs1042522 which is a non-synonymous amino acid substitution (Arg72Pro), showed a 6% increase in prevalence of type 2 diabetes per minor allele suggesting that this amino acid polymorphism may be a novel type 2 diabetes susceptibility variant. Furthermore, quantitative trait analysis in the Danish Inter99 showed a nominal association with increased fasting plasma glucose levels during an OGTT in carriers of the minor diabetogenic Arg-variant.

*TP53* encodes the tumor suppressor protein p53, which is known to be involved in cell-cycle control, apoptosis and maintenance of genetic stability, thereby protecting the organism from cellular damage [13]. P53 is expressed in all tissues, but at very low levels under normal conditions. It is also expressed in the pancreas and Arg72Pro which is located in the putative SH3 binding domain of p53 is by applying PolyPhen2® [14] predicted to have a probably damaging impact on the structure of the protein. Similarly, it has been shown, that the Arg72 variant increases p53 ability to induce apoptosis whereas the Pro-encoding allele exhibits a lower apoptotic potential [15]. An increased apoptotic rate of the pancreatic beta-cells might lead to an impairment of insulin secretion and possibly to an elevation of plasma glucose. This hypothesis was, however, not supported by the quantitative studies, since we did not find decreased serum insulin levels during an OGTT. Still, we do not have sufficient statistical power to detect low-impact genetic effects. Since rs1042522 is not in high LD with other HapMap-genotyped variants in the *TP53* region, the Arg72Pro variant possibly represents the diabetogenic variant in this locus. In 2010 a variant (rs1799941) in *SHBG*, which is very close to *TP53* (<40kb), showed association with type 2 diabetes [16], [17]. We found no linkage in our study population between rs1042522 and rs1799941 (r^2^ = 0.01), so we believe that these associations with type 2 diabetes represents independent signals. The Arg72 polymorphism has previously been reported to associate with increased risk of several forms of cancers [18]–[20].

The DIAGRAM+ Consortium data used in the combined meta-analysis include the FUSION study on which the initial report was based [2]. We were not technically able to exclude these data from the meta-analysis which may therefore be somewhat biased. Yet, given the fact that FUSION data are a minor part of the meta-analysis and that effect estimates of Danish and DIAGRAM+ data are virtually similar (OR 1.060 and 1.065, respectively) we argue that the current analyses are robust. We were only able to perform additive analyses when using DIAGRAM+ data. We could not make a complete replication of the primary findings according to genetic model, and there is a possibility that this could have strengthened the association with type 2 diabetes for the investigated variants.

The remaining variants could represent genuine novel low-impact type 2 diabetes variants; however current data do not support this notion and substantial sample sizes are needed to clarify a possible association.

The strongest impact on type 2 diabetes when exclusively analysing the Danish data was found for the *ENPP1* rs2021966 variant (OR = 1.15 95% CI 1.03–1.28, *P* = 0.015). After correction for multiple testing this variant was, however, not associated with type 2 diabetes in the present meta-analysis including 55,521 individuals (OR = 1.05, 1.01–1.1, *P* = 0.012). Variation in *ENPP1* has previously been associated with obesity, type 2 diabetes and insulin resistance [21], [22]. Interestingly, the minor Q allele of a K121Q (lysine to glutamine) variant (rs1044498) in *ENPP1* has been shown to influence the function of the protein by inhibiting insulin signalling by reducing receptor function and subsequent downstream signalling more effectively than the major K allele [22]. A meta-analysis of more than 40.000 individuals showed that *ENPP1* K121Q increases risk of type 2 diabetes under a recessive model; an effect that may be modulated by BMI [23]. There is low linkage disequilibrium between rs1044498) in *ENPP1* and the two *ENPP1* related variants investigated in the present study (r^2^<0.3 and D′<0.6 in Inter99).Because of the low impact on type 2 diabetes, and therefore presumably low impact on intermediary phenotypes for these *ENPP1* intronic variants, we need larger well-characterised study populations to clarify their potential underlying metabolic influence.

In conclusion, we demonstrate that *TP53* rs1042522 is associated with type 2 diabetes in a meta-analysis of 55,521 Europeans suggesting that *TP53* may be a novel type 2 diabetes susceptibility locus.

## Supporting Information

Table S1 **Members of the DIAGRAM consortium.**
(DOC)

Table S2 **Clinical characteristics of study populations.**
(DOC)

Table S3 **Anthropometric and metabolic characteristics of middle-aged treatment-naive Danish Inter99 participants stratified according to genotype of** ***ENPP1*** **rs2021966.**
(DOC)

Table S4 **Anthropometric and metabolic characteristics of middle-aged treatment-naive Danish Inter99 participants stratified according to genotype of** ***NRF1*** **rs1882095.**
(DOC)

Table S5 **Anthropometric and metabolic characteristics of middle-aged treatment-naive Danish Inter99 participants stratified according to genotype of** ***ENPP1*** **rs858341.**
(DOC)

Table S6 **Anthropometric and metabolic characteristics of middle-aged treatment-naive Danish Inter99 participants stratified according to genotype of** ***RAPGEF1*** **rs4740283.**
(DOC)

Table S7 **Anthropometric and metabolic characteristics of middle-aged treatment-naive Danish Inter99 participants stratified according to genotype** ***FOXC2*** **rs4843165.**
(DOC)

Table S8 **Anthropometric and metabolic characteristics of middle-aged treatment-naive Danish Inter99 participants stratified according to genotype of** ***SLC2A2*** **rs10513684.**
(DOC)

Table S9 **Anthropometric and metabolic characteristics of middle-aged treatment-naive Danish Inter99 participants stratified according to genotype of** ***SLC2A2*** **rs5400.**
(DOC)

Table S10 **Anthropometric and metabolic characteristics of middle-aged treatment-naive Danish Inter99 participants stratified according to genotype of** ***SLC2A4*** **rs222852.**
(DOC)
