# Supplementary material for: Studies of the Association of Arg72Pro of Tumor Suppressor Protein p53 with Type 2 Diabetes in a Combined Analysis of 55,521 Europeans
Source: PLoS One. 2011 Jan 20;6(1):e15813. doi: 10.1371/journal.pone.0015813 (PMC3024396; doi:10.1371/journal.pone.0015813)
Supplement: Table S2 — Clinical characteristics of study populations. (DOC) [file pone.0015813.s002.doc]

| **Study group 1 – Inter99** | **All** |  | **Normal glucose tolerant**  **(NGT)** | **Impaired glucose tolerance**  **(IGT)** | **Impaired fasting glycaemia**  **(IFG)** | **Treatment naive diabetics** | **Type 2 diabetes**  **patients** |
| --- | --- | --- | --- | --- | --- | --- | --- |
| *N* | 5999 |  | 4458 | 493 | 680 | 251 | 117 |
| Men/women | 2986/3013 |  | 2067/2391 | 363/130 | 335/345 | 160/91 | 61/56 |
| Age (years) | 46 ± 8 |  | 45 ± 8 | 49 ± 7 | 48 ± 8 | 51 ± 7 | 50.7 ± 7.6 |
| BMI (kg/m^2^) | 26.3 ± 4.6 |  | 25.5 ± 4.1 | 27.9 ± 4.6 | 28.1 ± 5.1 | 30.2 ± 5.8 | 29.9 ± 5.3 |
| HbA1c (%) | 5.9 ± 0.6 |  | 5.8 ± 0.4 | 6.0 ± 0.4 | 5.9 ± 0.4 | 6.6 ± 1.4 | 8.1 ± 1.8 |
| Fasting plasma glucose (mmol/l) | 5.6 ± 1.1 |  | 5.3 ± 0.4 | 6.3 ± 0.2 | 5.7 ± 0.5 | 7.5 ± 2.3 | 9.9 ± 3.9 |
| Waist (cm) | 87 ± 13 |  | 84 ± 12 | 93 ± 12 | 92 ± 14 | 98 ± 14 | 99 ± 14 |
| **Study group 2**  **Steno Diabetes Center**  **Unrelated diabetics** | **All** | **Non type 2 diabetics** |  |  |  |  | **Type 2 diabetes**  **patients** |
| *N* | 2013 | 392 |  |  |  |  | 1621 |
| Men/women | 1229/784 | 230/162 |  |  |  |  | 999/622 |
| Age (years) | 62 ± 11 | 60 ± 12 |  |  |  |  | 62 ± 11 |
| BMI (kg/m^2^) | 30.0 ± 5.6 | 28.8 ± 5.3 |  |  |  |  | 30.3 ± 5.6 |
| HbA1c (%) | NA | NA |  |  |  |  | NA |
| Fasting plasma glucose (mmol/l) | NA | NA |  |  |  |  | NA |
| Waist (cm) | 104 ± 15 | 99 ± 14 |  |  |  |  | 105 ± 15 |
| **Study group 3**  **Steno Diabetes Center**  **Population based** | **All** |  | **Normal glucose tolerant**  **(NGT)** |  |  | **Treatment naive diabetics** | **Type 2 diabetes**  **patients** |
| *N* | 562 |  | 515 |  |  | 28 | 19 |
| Men/women | 333/335 |  | 241/274 |  |  | 19/9 | 12/7 |
| Age (years) | 57 ± 10 |  | 57 ± 10 |  |  | 60 ± 8 | 61 ± 8 |
| BMI (kg/m^2^) | 26.3 ± 4.2 |  | 25.8 ± 3.7 |  |  | 27.8 ± 4.6 | 32.4 ± 6.4 |
| HbA1c (%) | 6.0 ± 0.8 |  | 5.8 ± 0.5 |  |  | 7.2 ± 1.5 | 9.1 ± 1.9 |
| Fasting plasma glucose (mmol/l) | 5.4 ± 1.3 |  | 5.1 ± 0.4 |  |  | 7.7 ± 2.3 | 10.3 ± 3.5 |
| Waist (cm) | 88 ± 12 |  | 86 ± 11 |  |  | 95 ± 13 | 101 ± 14 |
| **Study group 4**  **The ADDITION cohort** | **All** | **Non-diabetics** |  |  |  | **Treatment naive type 2 diabetics** |  |
| *N* | 1583 | 7 |  |  |  | 1576 |  |
| Men/women | 902/681 | 3/4 |  |  |  | 899/677 |  |
| Age (years) | 60 ± 7 | 58 ± 10 |  |  |  | 60 ± 7 |  |
| BMI (kg/m^2^) | 31.1 ± 5.4 | 31.4 ± 5.3 |  |  |  | 31.1 ± 5.4 |  |
| HbA1c (%) | NA | NA |  |  |  | NA |  |
| Fasting plasma glucose (mmol/l) | NA | NA |  |  |  | NA |  |
| Waist (cm) | 104 ± 14 | 100 ± 13 |  |  |  | 104 ± 14 |  |

**Table S2: Clinical characteristics of study populations**

Data are mean +/- standard deviation. NA not available.
