# Supplementary material for: Studies of the Association of Arg72Pro of Tumor Suppressor Protein p53 with Type 2 Diabetes in a Combined Analysis of 55,521 Europeans
Source: PLoS One. 2011 Jan 20;6(1):e15813. doi: 10.1371/journal.pone.0015813 (PMC3024396; doi:10.1371/journal.pone.0015813)
Supplement: Table S3 — Anthropometric and metabolic characteristics of middle-aged treatment-naive Danish Inter99 participants stratified according to genotype of ENPP1 rs2021966. (DOC) [file pone.0015813.s003.doc]

**Table S3** Anthropometric and metabolic characteristics of middle-aged treatment-naive Danish Inter99 participants stratified according to genotype of *ENPP1* rs2021966

| ***ENPP1* rs2021966** | **TT** | **TA** | **AA** | ***P*** |
| --- | --- | --- | --- | --- |
| *n* (men/women) | 1576(794/782) | 2750(1365/1385) | 1304(650/654) |  |
| Age (years) | 46 ± 8 | 46 ± 8 | 46 ± 8 |  |
| BMI (kg/m^2^) | 26.1 ± 4.4 | 26.3 ± 4.7 | 26.1 ± 4.4 | 0.75 |
| Waist-to-hip ratio | 0.86 ± 0.09 | 0.85 ± 0.09 | 0.86 ± 0.09 | 0.18 |
| waist (cm) | 86 ± 13 | 87 ± 13 | 86 ± 13 | 0.85 |
| **Plasma glucose** |  |  |  |  |
| Fasting (mmol/l) | 5.5 ± 0.7 | 5.5 ± 0.8 | 5.6 ± 0.9 | 0.75 |
| 30-min post-OGTT (mmol/l) | 8.8 ± 1.9 | 8.7 ± 1.8 | 8.7 ± 2 | 0.019 |
| 120-min post-OGTT (mmol/l) | 6.3 ± 2.1 | 6.2 ± 2 | 6.2 ± 2.4 | 0.25 |
| Post-OGTT AUC (min⋅mmol/l) | 227 ± 141 | 219 ± 132 | 216 ± 138 | 0.015 |
| **Serum insulin** |  |  |  |  |
| Fasting (pmol/l) | 42 ± 26 | 42 ± 28 | 42 ± 27 | 0.45 |
| 30-min post-OGTT (pmol/l) | 290 ± 185 | 293 ± 184 | 288 ± 180 | 0.38 |
| 120-min post-OGTT (pmol/l) | 220 ± 216 | 220 ± 220 | 205 ± 188 | 0.055 |
| Post-OGTT AUC (min⋅pmol/l) | 22994 ± 15921 | 23195 ± 16466 | 22175 ± 14646 | 0.079 |
| HOMA-IR (mmol/l⋅pmol/l) | 10.5 ± 7.6 | 10.6 ± 8.2 | 10.6 ± 7.8 | 0.48 |
| Insulinogenic index (pmol×pmol^−1^) | 29 ± 20 | 30 ± 20 | 29 ± 19 | 0.61 |
| BIGTT-SI | 9.2 ± 4 | 9.2 ± 4 | 9.3 ± 4 | 0.69 |
| BIGTT-AIR | 1796 ± 1003 | 1870 ± 1066 | 1852 ± 1151 | 0.11 |
| **Fasting serum lipids** |  |  |  |  |
| Triglyceride (mmol/l) | 1.4 ± 1.8 | 1.3 ± 1.3 | 1.3 ± 0.8 | 0.11 |
| Total cholesterol (mmol/l) | 5.5 ± 1.1 | 5.5 ± 1.1 | 5.6 ± 1.1 | 0.94 |
| HDL-cholesterol (mmol/l) | 1.4 ± 0.4 | 1.4 ± 0.4 | 1.5 ± 0.4 | 0.4 |

Data are mean +/- standard deviation. Values of serum insulin, values derived from insulin variables, and values of serum triglyceride were logarithmically transformed before statistical analysis. Calculated *P* values were adjusted for age, sex, and for BMI (except BMI, waist-to-hip and waist), and were calculated assuming an additive model. HOMA-IR was calculated as fasting plasma glucose (mmol/l) multiplied by fasting serum insulin (pmol/l) and divided by 22.5. AUC, area under the curve.
