# Supplementary material for: Studies of the Association of Arg72Pro of Tumor Suppressor Protein p53 with Type 2 Diabetes in a Combined Analysis of 55,521 Europeans
Source: PLoS One. 2011 Jan 20;6(1):e15813. doi: 10.1371/journal.pone.0015813 (PMC3024396; doi:10.1371/journal.pone.0015813)
Supplement: Table S4 — Anthropometric and metabolic characteristics of middle-aged treatment-naive Danish Inter99 participants stratified according to genotype of NRF1 rs1882095. (DOC) [file pone.0015813.s004.doc]

| ***NRF1* rs1882095** | **CC** | **CT** | **TT** | ***P*** |
| --- | --- | --- | --- | --- |
| *n* (men/women) | 2406(1215/1191) | 2612(1296/1316) | 705(343/362) |  |
| Age (years) | 46 ± 8 | 46 ± 8 | 46 ± 8 |  |
| BMI (kg/m^2^) | 26.3 ± 4.5 | 26.1 ± 4.5 | 26.2 ± 4.5 | 0.26 |
| Waist-to-hip ratio | 0.86 ± 0.09 | 0.85 ± 0.08 | 0.86 ± 0.09 | 0.56 |
| waist (cm) | 87 ± 13 | 86 ± 13 | 86 ± 13 | 0.25 |
| **Plasma glucose** |  |  |  |  |
| Fasting (mmol/l) | 5.6 ± 0.8 | 5.5 ± 0.8 | 5.5 ± 0.8 | 0.3 |
| 30-min post-OGTT (mmol/l) | 8.7 ± 1.9 | 8.7 ± 1.9 | 8.7 ± 2 | 0.79 |
| 120-min post-OGTT (mmol/l) | 6.2 ± 2.1 | 6.2 ± 2.2 | 6.3 ± 2.2 | 0.65 |
| Post-OGTT AUC (min⋅mmol/l) | 222 ± 133 | 218 ± 136 | 226 ± 139 | 0.5 |
| **Serum insulin** |  |  |  |  |
| Fasting (pmol/l) | 43 ± 28 | 41 ± 28 | 42 ± 28 | 0.38 |
| 30-min post-OGTT (pmol/l) | 290 ± 183 | 289 ± 183 | 296 ± 183 | 0.4 |
| 120-min post-OGTT (pmol/l) | 221 ± 212 | 211 ± 207 | 221 ± 224 | 0.41 |
| Post-OGTT AUC (min⋅pmol/l) | 23003 ± 16028 | 22612 ± 15332 | 23472 ± 16823 | 0.54 |
| HOMA-IR (mmol/l⋅pmol/l) | 10.8 ± 7.9 | 10.4 ± 7.9 | 10.5 ± 8.9 | 0.29 |
| Insulinogenic index (pmol×pmol^−1^) | 29 ± 19 | 29 ± 20 | 30 ± 19 | 0.27 |
| BIGTT-SI | 9.0 ± 4 | 9.4 ± 4 | 9.3 ± 4 | 0.056 |
| BIGTT-AIR | 1849 ± 1066 | 1829 ± 1089 | 1880 ± 995 | 0.93 |
| **Fasting serum lipids** |  |  |  |  |
| Triglyceride (mmol/l) | 1.3 ± 1.5 | 1.3 ± 1 | 1.4 ± 1.7 | 0.36 |
| Total cholesterol (mmol/l) | 5.5 ± 1 | 5.5 ± 1.1 | 5.6 ± 1.1 | 0.09 |
| HDL-cholesterol (mmol/l) | 1.4 ± 0.4 | 1.4 ± 0.4 | 1.4 ± 0.4 | 0.78 |

**Table S4** Anthropometric and metabolic characteristics of middle-aged treatment-naive Danish Inter99 participants stratified according to genotype of *NRF1* rs1882095

Data are mean +/- standard deviation. Values of serum insulin, values derived from insulin variables, and values of serum triglyceride were logarithmically transformed before statistical analysis. Calculated *P* values were adjusted for age, sex, and for BMI (except BMI, waist-to-hip and waist), and were calculated assuming an additive model. HOMA-IR was calculated as fasting plasma glucose (mmol/l) multiplied by fasting serum insulin (pmol/l) and divided by 22.5. AUC, area under the curve.
