# Supplementary material for: Studies of the Association of Arg72Pro of Tumor Suppressor Protein p53 with Type 2 Diabetes in a Combined Analysis of 55,521 Europeans
Source: PLoS One. 2011 Jan 20;6(1):e15813. doi: 10.1371/journal.pone.0015813 (PMC3024396; doi:10.1371/journal.pone.0015813)
Supplement: Table S5 — Anthropometric and metabolic characteristics of middle-aged treatment-naive Danish Inter99 participants stratified according to genotype of ENPP1 rs858341. (DOC) [file pone.0015813.s005.doc]

**Table S5** Anthropometric and metabolic characteristics of middle-aged treatment-naive Danish Inter99 participants stratified according to genotype of *ENPP1* rs858341

| ***ENPP1* rs858341** | **AA** | **AG** | **GG** | ***P*** |
| --- | --- | --- | --- | --- |
| *n* (men/women) | 2295(1118/1177) | 2649(1327/1322) | 833(429/404) |  |
| Age (years) | 46 ± 8 | 46 ± 8 | 46 ± 8 |  |
| BMI (kg/m^2^) | 26.2 ± 4.4 | 26.2 ± 4.8 | 26.2 ± 4.1 | 0.62 |
| Waist-to-hip ratio | 0.85 ± 0.09 | 0.86 ± 0.09 | 0.86 ± 0.08 | 0.29 |
| waist (cm) | 86 ± 13 | 86 ± 14 | 87 ± 13 | 0.80 |
| **Plasma glucose** |  |  |  |  |
| Fasting (mmol/l) | 5.5 ± 0.9 | 5.5 ± 0.8 | 5.6 ± 0.6 | 0.91 |
| 30-min post-OGTT (mmol/l) | 8.6 ± 1.9 | 8.7 ± 1.9 | 8.9 ± 1.9 | 0.0022 |
| 120-min post-OGTT (mmol/l) | 6.2 ± 2.2 | 6.2 ± 2.1 | 6.2 ± 2 | 0.25 |
| Post-OGTT AUC (min⋅mmol/l) | 215 ± 133 | 222 ± 135 | 230 ± 143 | 0.0022 |
| **Serum insulin** |  |  |  |  |
| Fasting (pmol/l) | 42 ± 28 | 42 ± 28 | 42 ± 26 | 0.99 |
| 30-min post-OGTT (pmol/l) | 288 ± 182 | 290 ± 187 | 297 ± 181 | 0.054 |
| 120-min post-OGTT (pmol/l) | 213 ± 209 | 219 ± 212 | 222 ± 217 | 0.073 |
| Post-OGTT AUC (min⋅pmol/l) | 22484 ± 15445 | 23027 ± 16410 | 23557 ± 15448 | 0.012 |
| HOMA-IR (mmol/l⋅pmol/l) | 10.6 ± 7.9 | 10.5 ± 8.3 | 10.5 ± 7.4 | 0.99 |
| Insulinogenic index (pmol×pmol^−1^) | 29 ± 19 | 29 ± 20 | 30 ± 20 | 0.21 |
| BIGTT-SI | 9.2 ± 4 | 9.3 ± 4 | 9.0 ± 4 | 0.21 |
| BIGTT-AIR | 1838 ± 1063 | 1859 ± 1110 | 1833 ± 1069 | 0.54 |
| **Fasting serum lipids** |  |  |  |  |
| Triglyceride (mmol/l) | 1.3 ± 0.9 | 1.4 ± 1.8 | 1.3 ± 0.8 | 0.05 |
| Total cholesterol (mmol/l) | 5.5 ± 1.1 | 5.5 ± 1.1 | 5.5 ± 1.1 | 0.21 |
| HDL-cholesterol (mmol/l) | 1.4 ± 0.4 | 1.4 ± 0.4 | 1.4 ± 0.4 | 0.77 |

Data are mean +/- standard deviation. Values of serum insulin, values derived from insulin variables, and values of serum triglyceride were logarithmically transformed before statistical analysis. Calculated *P* values were adjusted for age, sex, and for BMI (except BMI, waist-to-hip and waist), and were calculated assuming an additive model. HOMA-IR was calculated as fasting plasma glucose (mmol/l) multiplied by fasting serum insulin (pmol/l) and divided by 22.5. AUC, area under the curve.
