# Supplementary material for: Studies of the Association of Arg72Pro of Tumor Suppressor Protein p53 with Type 2 Diabetes in a Combined Analysis of 55,521 Europeans
Source: PLoS One. 2011 Jan 20;6(1):e15813. doi: 10.1371/journal.pone.0015813 (PMC3024396; doi:10.1371/journal.pone.0015813)
Supplement: Table S6 — Anthropometric and metabolic characteristics of middle-aged treatment-naive Danish Inter99 participants stratified according to genotype of RAPGEF1 rs4740283. (DOC) [file pone.0015813.s006.doc]

**Table S6** Anthropometric and metabolic characteristics of middle-aged treatment-naive Danish Inter99 participants stratified according to genotype of *RAPGEF1* rs4740283

| ***RAPGEF1* rs4740283** | **AA** | **AG** | **GG** | ***P*** |
| --- | --- | --- | --- | --- |
| *n* (men/women) | 4244(2095/2149) | 1397(709/688) | 114(59/55) |  |
| Age (years) | 46 ± 8 | 46 ± 8 | 45 ± 7 |  |
| BMI (kg/m^2^) | 26.3 ± 4.5 | 26 ± 4.5 | 25.3 ± 4.2 | 0.003 |
| Waist-to-hip ratio | 0.86 ± 0.09 | 0.85 ± 0.09 | 0.84 ± 0.09 | 0.0073 |
| waist (cm) | 87 ± 13 | 86 ± 13 | 84 ± 13 | 0.0037 |
| **Plasma glucose** |  |  |  |  |
| Fasting (mmol/l) | 5.5 ± 0.8 | 5.5 ± 0.8 | 5.5 ± 0.6 | 0.77 |
| 30-min post-OGTT (mmol/l) | 8.7 ± 1.9 | 8.7 ± 1.9 | 8.5 ± 1.5 | 0.7 |
| 120-min post-OGTT (mmol/l) | 6.2 ± 2.1 | 6.2 ± 2.1 | 6.2 ± 1.8 | 0.94 |
| Post-OGTT AUC (min⋅mmol/l) | 221 ± 135 | 220 ± 137 | 212 ± 117 | 0.89 |
| **Serum insulin** |  |  |  |  |
| Fasting (pmol/l) | 42 ± 28 | 42 ± 29 | 36 ± 20 | 0.8 |
| 30-min post-OGTT (pmol/l) | 291 ± 184 | 291 ± 183 | 260 ± 138 | 0.61 |
| 120-min post-OGTT (pmol/l) | 216 ± 208 | 223 ± 227 | 189 ± 167 | 0.46 |
| Post-OGTT AUC (min⋅pmol/l) | 22797 ± 15662 | 23214 ± 16637 | 20603 ± 12643 | 0.26 |
| HOMA-IR (mmol/l⋅pmol/l) | 10.6 ± 8 | 10.7 ± 8.3 | 8.9 ± 5.5 | 0.78 |
| Insulinogenic index (pmol×pmol^−1^) | 29 ± 20 | 29 ± 19 | 27 ± 17 | 0.46 |
| BIGTT-SI | 9.2 ± 4 | 9.2 ± 4 | 10.0 ± 4 | 0.21 |
| BIGTT-AIR | 1853 ± 1086 | 1828 ± 1040 | 1681 ± 590 | 0.077 |
| **Fasting serum lipids** |  |  |  |  |
| Triglyceride (mmol/l) | 1.4 ± 1.5 | 1.3 ± 0.9 | 1.2 ± 0.6 | 0.41 |
| Total cholesterol (mmol/l) | 5.5 ± 1.1 | 5.5 ± 1.1 | 5.5 ± 1.2 | 0.76 |
| HDL-cholesterol (mmol/l) | 1.4 ± 0.4 | 1.4 ± 0.4 | 1.4 ± 0.4 | 0.89 |

Data are mean +/- standard deviation. Values of serum insulin, values derived from insulin variables, and values of serum triglyceride were logarithmically transformed before statistical analysis. Calculated *P* values were adjusted for age, sex, and for BMI (except BMI, waist-to-hip and waist), and were calculated assuming an additive model. HOMA-IR was calculated as fasting plasma glucose (mmol/l) multiplied by fasting serum insulin (pmol/l) and divided by 22.5. AUC, area under the curve.
