# Supplementary material for: Studies of the Association of Arg72Pro of Tumor Suppressor Protein p53 with Type 2 Diabetes in a Combined Analysis of 55,521 Europeans
Source: PLoS One. 2011 Jan 20;6(1):e15813. doi: 10.1371/journal.pone.0015813 (PMC3024396; doi:10.1371/journal.pone.0015813)
Supplement: Table S7 — Anthropometric and metabolic characteristics of middle-aged treatment-naive Danish Inter99 participants stratified according to genotype FOXC2 rs4843165. (DOC) [file pone.0015813.s007.doc]

**Table S7** Anthropometric and metabolic characteristics of middle-aged treatment-naive Danish Inter99 participants stratified according to genotype *FOXC2* rs4843165

| **FOXC2 rs4843165** | **CC** | **CT** | **TT** | ***P*** |
| --- | --- | --- | --- | --- |
| *n* (men/women) | 3128(1546/1582) | 2167(1092/1075) | 429(211/218) |  |
| Age (years) | 46 ± 8 | 46 ± 8 | 46 ± 8 |  |
| BMI (kg/m^2^) | 26.2 ± 4.6 | 26.2 ± 4.5 | 26.1 ± 4.4 | 0.94 |
| Waist-to-hip ratio | 0.86 ± 0.09 | 0.85 ± 0.09 | 0.85 ± 0.09 | 0.23 |
| waist (cm) | 87 ± 13 | 86 ± 13 | 86 ± 13 | 0.49 |
| **Plasma glucose** |  |  |  |  |
| Fasting (mmol/l) | 5.5 ± 0.8 | 5.5 ± 0.8 | 5.6 ± 0.9 | 0.82 |
| 30-min post-OGTT (mmol/l) | 8.7 ± 1.9 | 8.7 ± 1.9 | 8.7 ± 1.8 | 0.66 |
| 120-min post-OGTT (mmol/l) | 6.2 ± 2.2 | 6.2 ± 2.1 | 6.1 ± 1.9 | 0.59 |
| Post-OGTT AUC (min⋅mmol/l) | 221 ± 137 | 220 ± 135 | 218 ± 128 | 0.94 |
| **Serum insulin** |  |  |  |  |
| Fasting (pmol/l) | 42 ± 28 | 42 ± 28 | 41 ± 27 | 0.17 |
| 30-min post-OGTT (pmol/l) | 291 ± 188 | 292 ± 179 | 283 ± 175 | 0.3 |
| 120-min post-OGTT (pmol/l) | 216 ± 205 | 220 ± 226 | 204 ± 187 | 0.44 |
| Post-OGTT AUC (min⋅pmol/l) | 22937 ± 15999 | 23023 ± 15980 | 21918 ± 14307 | 0.26 |
| HOMA-IR (mmol/l⋅pmol/l) | 10.6 ± 7.9 | 10.5 ± 8.4 | 10.3 ± 7.4 | 0.19 |
| Insulinogenic index (pmol×pmol^−1^) | 29 ± 20 | 30 ± 20 | 28 ± 19 | 0.31 |
| BIGTT-SI | 9.2 ± 4 | 9.3 ± 4 | 9.4 ± 4 | 0.61 |
| **Fasting serum lipids** |  |  |  |  |
| Triglyceride (mmol/l) | 1.3 ± 1 | 1.3 ± 1.2 | 1.5 ± 3.1 | 0.26 |
| Total cholesterol (mmol/l) | 5.5 ± 1.1 | 5.5 ± 1.1 | 5.5 ± 1.1 | 0.72 |
| HDL-cholesterol (mmol/l) | 1.4 ± 0.4 | 1.4 ± 0.4 | 1.4 ± 0.4 | 0.91 |

Data are mean +/- standard deviation. Values of serum insulin, values derived from insulin variables, and values of serum triglyceride were logarithmically transformed before statistical analysis. Calculated *P* values were adjusted for age, sex, and for BMI (except BMI, waist-to-hip and waist), and were calculated assuming an additive model. HOMA-IR was calculated as fasting plasma glucose (mmol/l) multiplied by fasting serum insulin (pmol/l) and divided by 22.5. AUC, area under the curve.
