# Supplementary material for: Studies of the Association of Arg72Pro of Tumor Suppressor Protein p53 with Type 2 Diabetes in a Combined Analysis of 55,521 Europeans
Source: PLoS One. 2011 Jan 20;6(1):e15813. doi: 10.1371/journal.pone.0015813 (PMC3024396; doi:10.1371/journal.pone.0015813)
Supplement: Table S8 — Anthropometric and metabolic characteristics of middle-aged treatment-naive Danish Inter99 participants stratified according to genotype of SLC2A2 rs10513684. (DOC) [file pone.0015813.s008.doc]

**Table S8** Anthropometric and metabolic characteristics of middle-aged treatment-naive Danish Inter99 participants stratified according to genotype of *SLC2A2* rs10513684

| ***SLC2A2* rs10513684** | **CC** | **CT** | **TT** | ***P*** |
| --- | --- | --- | --- | --- |
| *n* (men/women) | 5149(2579/2570) | 604(291/313) | 19(6/13) |  |
| Age (years) | 46 ± 8 | 46 ± 8 | 48 ± 8 |  |
| BMI (kg/m^2^) | 26.2 ± 4.5 | 26 ± 4.6 | 26.8 ± 5.2 | 0.68 |
| Waist-to-hip ratio | 0.86 ± 0.09 | 0.85 ± 0.08 | 0.84 ± 0.1 | 0.84 |
| waist (cm) | 87 ± 13 | 86 ± 13 | 88 ± 18 | 0.97 |
| **Plasma glucose** |  |  |  |  |
| Fasting (mmol/l) | 5.5 ± 0.8 | 5.5 ± 0.6 | 5.5 ± 0.7 | 0.06 |
| 30-min post-OGTT (mmol/l) | 8.7 ± 1.9 | 8.7 ± 1.8 | 9.1 ± 1.6 | 0.2 |
| 120-min post-OGTT (mmol/l) | 6.2 ± 2.2 | 6.1 ± 1.9 | 6.4 ± 2.2 | 0.31 |
| Post-OGTT AUC (min⋅mmol/l) | 220 ± 136 | 220 ± 132 | 257 ± 148 | 0.34 |
| **Serum insulin** |  |  |  |  |
| Fasting (pmol/l) | 42 ± 28 | 42 ± 27 | 40 ± 27 | 0.72 |
| 30-min post-OGTT (pmol/l) | 291 ± 183 | 289 ± 188 | 261 ± 181 | 0.84 |
| 120-min post-OGTT (pmol/l) | 218 ± 213 | 207 ± 195 | 235 ± 234 | 0.56 |
| Post-OGTT AUC (min⋅pmol/l) | 22962 ± 15879 | 22116 ± 16104 | 22090 ± 17325 | 0.33 |
| HOMA-IR (mmol/l⋅pmol/l) | 10.6 ± 8.1 | 10.4 ± 7.2 | 10.2 ± 7.7 | 0.98 |
| Insulinogenic index (pmol×pmol^−1^) | 29 ± 20 | 29 ± 19 | 24 ± 15 | 0.3 |
| BIGTT-SI | 9.2 ± 4 | 9.4 ± 4 | 9.8 ± 6 | 0.19 |
| BIGTT-AIR | 1846 ± 1085 | 1844 ± 1072 | 1615 ± 670 | 0.43 |
| **Fasting serum lipids** |  |  |  |  |
| Triglyceride (mmol/l) | 1.3 ± 1.4 | 1.3 ± 0.9 | 1.7 ± 1.5 | 0.16 |
| Total cholesterol (mmol/l) | 5.5 ± 1.1 | 5.5 ± 1.1 | 5.3 ± 1.4 | 0.55 |
| HDL-cholesterol (mmol/l) | 1.4 ± 0.4 | 1.4 ± 0.4 | 1.5 ± 0.6 | 0.94 |

Data are mean +/- standard deviation. Values of serum insulin, values derived from insulin variables, and values of serum triglyceride were logarithmically transformed before statistical analysis. Calculated *P* values were adjusted for age, sex, and for BMI (except BMI, waist-to-hip and waist), and were calculated assuming an additive model. HOMA-IR was calculated as fasting plasma glucose (mmol/l) multiplied by fasting serum insulin (pmol/l) and divided by 22.5. AUC, area under the curve.
