# Supplementary material for: Studies of the Association of Arg72Pro of Tumor Suppressor Protein p53 with Type 2 Diabetes in a Combined Analysis of 55,521 Europeans
Source: PLoS One. 2011 Jan 20;6(1):e15813. doi: 10.1371/journal.pone.0015813 (PMC3024396; doi:10.1371/journal.pone.0015813)
Supplement: Table S9 — Anthropometric and metabolic characteristics of middle-aged treatment-naive Danish Inter99 participants stratified according to genotype of SLC2A2 rs5400. (DOC) [file pone.0015813.s009.doc]

**Table S9** Anthropometric and metabolic characteristics of middle-aged treatment-naive Danish Inter99 participants stratified according to genotype of *SLC2A2* rs5400

| ***SLC2A2* rs5400** | **CC** | **CT** | **TT** | ***P*** |
| --- | --- | --- | --- | --- |
| *n* (men/women) | 4335(2172/2163) | 1324(664/660) | 132(50/82) |  |
| Age (years) | 46 ± 8 | 46 ± 8 | 46 ± 8 |  |
| BMI (kg/m^2^) | 26.2 ± 4.6 | 26.1 ± 4.5 | 26.2 ± 4.2 | 0.77 |
| Waist-to-hip ratio | 0.86 ± 0.09 | 0.85 ± 0.08 | 0.84 ± 0.09 | 0.52 |
| waist (cm) | 87 ± 13 | 86 ± 13 | 85 ± 13 | 0.46 |
| **Plasma glucose** |  |  |  |  |
| Fasting (mmol/l) | 5.6 ± 0.8 | 5.5 ± 0.7 | 5.5 ± 0.7 | 0.45 |
| 30-min post-OGTT (mmol/l) | 8.7 ± 1.9 | 8.7 ± 1.8 | 8.7 ± 1.8 | 0.18 |
| 120-min post-OGTT (mmol/l) | 6.2 ± 2.1 | 6.2 ± 2.1 | 6.2 ± 2 | 0.66 |
| Post-OGTT AUC (min⋅mmol/l) | 221 ± 136 | 220 ± 135 | 224 ± 131 | 0.44 |
| **Serum insulin** |  |  |  |  |
| Fasting (pmol/l) | 42 ± 28 | 42 ± 28 | 42 ± 28 | 0.78 |
| 30-min post-OGTT (pmol/l) | 292 ± 185 | 287 ± 181 | 276 ± 156 | 0.3 |
| 120-min post-OGTT (pmol/l) | 220 ± 216 | 211 ± 202 | 209 ± 168 | 0.6 |
| Post-OGTT AUC (min⋅pmol/l) | 23093 ± 16063 | 22477 ± 15664 | 21775 ± 12790 | 0.41 |
| HOMA-IR (mmol/l⋅pmol/l) | 10.6 ± 7.9 | 10.5 ± 8.3 | 10.5 ± 8.5 | 0.65 |
| Insulinogenic index (pmol×pmol^−1^) | 29 ± 20 | 29 ± 19 | 27 ± 16 | 0.14 |
| BIGTT-SI | 9.2 ± 4 | 9.3 ± 4 | 9.5 ± 5 | 0.36 |
| BIGTT-AIR | 1850 ± 1068 | 1838 ± 1146 | 1734 ± 686 | 0.14 |
| **Fasting serum lipids** |  |  |  |  |
| Triglyceride (mmol/l) | 1.3 ± 1.4 | 1.3 ± 1.1 | 1.3 ± 0.8 | 0.57 |
| Total cholesterol (mmol/l) | 5.5 ± 1.1 | 5.6 ± 1.1 | 5.6 ± 1.2 | 0.02 |
| HDL-cholesterol (mmol/l) | 1.4 ± 0.4 | 1.5 ± 0.4 | 1.5 ± 0.5 | 0.02 |

Data are mean +/- standard deviation. Values of serum insulin, values derived from insulin variables, and values of serum triglyceride were logarithmically transformed before statistical analysis. Calculated *P* values were adjusted for age, sex, and for BMI (except BMI, waist-to-hip and waist), and were calculated assuming an additive model. HOMA-IR was calculated as fasting plasma glucose (mmol/l) multiplied by fasting serum insulin (pmol/l) and divided by 22.5. AUC, area under the curve.
