# Supplementary material for: Studies of the Association of Arg72Pro of Tumor Suppressor Protein p53 with Type 2 Diabetes in a Combined Analysis of 55,521 Europeans
Source: PLoS One. 2011 Jan 20;6(1):e15813. doi: 10.1371/journal.pone.0015813 (PMC3024396; doi:10.1371/journal.pone.0015813)
Supplement: Table S10 — Anthropometric and metabolic characteristics of middle-aged treatment-naive Danish Inter99 participants stratified according to genotype of SLC2A4 rs222852. (DOC) [file pone.0015813.s010.doc]

**Table S10** Anthropometric and metabolic characteristics of middle-aged treatment-naive Danish Inter99 participants stratified according to genotype of *SLC2A4* rs222852

| **SLC2A4 rs222852** | **AA** | **AG** | **GG** | ***P*** |
| --- | --- | --- | --- | --- |
| *n* (men/women) | 1948(953/995) | 2739(1392/1347) | 1066(520/546) |  |
| Age (years) | 46 ± 8 | 46 ± 8 | 46 ± 8 |  |
| BMI (kg/m^2^) | 26.3 ± 4.6 | 26.2 ± 4.5 | 26.1 ± 4.6 | 0.38 |
| Waist-to-hip ratio | 0.85 ± 0.09 | 0.86 ± 0.08 | 0.85 ± 0.08 | 0.39 |
| waist (cm) | 86 ± 13 | 87 ± 13 | 86 ± 13 | 0.46 |
| **Plasma glucose** |  |  |  |  |
| Fasting (mmol/l) | 5.5 ± 0.7 | 5.5 ± 0.8 | 5.5 ± 0.7 | 0.54 |
| 30-min post-OGTT (mmol/l) | 8.7 ± 1.8 | 8.7 ± 1.9 | 8.6 ± 1.8 | 0.39 |
| 120-min post-OGTT (mmol/l) | 6.2 ± 2 | 6.2 ± 2.2 | 6.2 ± 2.1 | 0.29 |
| Post-OGTT AUC (min⋅mmol/l) | 220 ± 136 | 221 ± 135 | 218 ± 134 | 0.85 |
| **Serum insulin** |  |  |  |  |
| Fasting (pmol/l) | 42 ± 28 | 42 ± 28 | 42 ± 28 | 0.68 |
| 30-min post-OGTT (pmol/l) | 294 ± 191 | 287 ± 180 | 290 ± 175 | 0.92 |
| 120-min post-OGTT (pmol/l) | 219 ± 215 | 217 ± 216 | 209 ± 184 | 0.99 |
| Post-OGTT AUC (min⋅pmol/l) | 23225 ± 16251 | 22676 ± 16031 | 22655 ± 14292 | 0.71 |
| HOMA-IR (mmol/l⋅pmol/l) | 10.6 ± 7.8 | 10.6 ± 8.2 | 10.4 ± 7.7 | 0.59 |
| Insulinogenic index (pmol×pmol^−1^) | 30 ± 20 | 29 ± 19 | 30 ± 20 | 1 |
| BIGTT-SI | 9.2 ± 4 | 9.2 ± 4 | 9.3 ± 4 | 0.55 |
| BIGTT-AIR | 1848 ± 1062 | 1825 ± 1027 | 1884 ± 1166 | 0.47 |
| **Fasting serum lipids** |  |  |  |  |
| Triglyceride (mmol/l) | 1.3 ± 0.9 | 1.3 ± 1.1 | 1.4 ± 2.3 | 0.48 |
| Total cholesterol (mmol/l) | 5.5 ± 1.1 | 5.5 ± 1.1 | 5.5 ± 1.1 | 0.39 |
| HDL-cholesterol (mmol/l) | 1.4 ± 0.4 | 1.4 ± 0.4 | 1.4 ± 0.4 | 0.67 |

Data are mean +/- standard deviation. Values of serum insulin, values derived from insulin variables, and values of serum triglyceride were logarithmically transformed before statistical analysis. Calculated *P* values were adjusted for age, sex, and for BMI (except BMI, waist-to-hip and waist), and were calculated assuming an additive model. HOMA-IR was calculated as fasting plasma glucose (mmol/l) multiplied by fasting serum insulin (pmol/l) and divided by 22.5. AUC, area under the curve.
